# Supplementary material for: Amphipathic Cell-Penetrating Peptide-Aided Delivery of Cas9 RNP for In Vitro Gene Editing and Correction
Source: Pharmaceutics. 2023 Oct 20;15(10):2500. doi: 10.3390/pharmaceutics15102500 (PMC10609989; doi:10.3390/pharmaceutics15102500)
Supplement: Supplementary file 1 [file pharmaceutics-15-02500-s001.zip › pharmaceutics-2641203-supplementary.pdf]

Supplementary Materials

Amphipathic Cell-Penetrating Peptide-Aided Delivery of Cas9 RNP for In Vitro Gene Editing and Correction

Mert Öktem, Enrico Mastrobattista and Olivier G. de Jong *

Department of Pharmaceutics, Utrecht Institute of Pharmaceutical Sciences (UIPS), Utrecht University, Universiteitsweg 99, 3584 CG Utrecht, The Netherlands; m.oktem@uu.nl (M.O); e.mastrobattista@uu.nl (E.M.)

* Correspondence: o.g.dejong@uu.nl

Table S1. Guide RNA sequences used in this work.

Target	sgRNA targeting sequence
Stoplight construct	GGACAGUACUCCGCUCGAGU
HDR stoplight construct	GCUUACUUGUACAGCUCGUCC
CCR5	UGACAUCAAUUAUUAUACAU
Non-target sgRNA	GUUAAUGUGGCUCUGGUUCU

Table S2. PCR primers used for amplification of the Stoplight and *CCR5* loci for both T7E1 and TIDE experiments.

Primer name	Sequence 5′- 3′
Stoplight Forward	GAAGGGCGAGATCAAGCAGA
Stoplight Reverse	GGTCTTGTAGTTGCCGTCGT
CCR5 Forward	CAACAGAGCCAAGCTCTCCAT
CCR5 Reverse	CCTGGGAGAGACGCAAACAC

Table S3. Single stranded HDR DNA template sequence. DNA mismatches and encoding the mutation, are specified with small letter.

HDR template name	Template sequence 5′-3′
HDR stoplight 81bp	ACGACGCCCGTGAAAAGCTCTTCACCCTTAGACACGGCTTgCTTGT ACAGCTCGTCCAaGCCGCCCGTAGAATCCCTGCCT
Alexa 647 labeled HDR template	/Alex647N/ ACGACGCCCGTGAAAAGCTCTTCACCCTTAGACACGGCTTGCTTG TACAGCTCGTCCAAGCCGCCCGTAGAATCCCTGCCT

Table S4. crRNA sequence used in this research.

Target	crRNA sequence
HPRT	CCUGACAAUCGAUAGGUACCGUUUUAGAGCUAUGCU

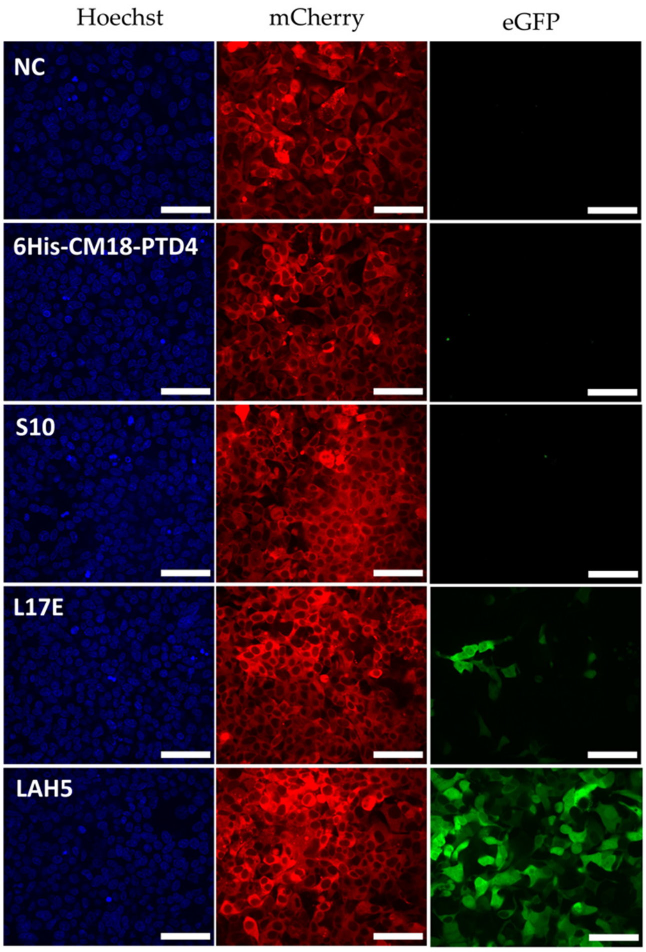

Figure S1. Screening of several amphipathic peptides to determine their capacity of delivering Cas9/RNPs. Confical microscopy images (60x) of HEK293T stoplight cells after treatment with 1:150 molar ratio of RNP:peptide at a RNP concentration of 20 nM. Red – mCherry, green – eGFP (Cas9 gene editing), blue – Hoechst 33342 nuclear dye, NC: Negative control. A schematic depiction of the fluorescent Stoplight reporter construct is depicted in Figure 5A. Scale bar represents 100 µm.

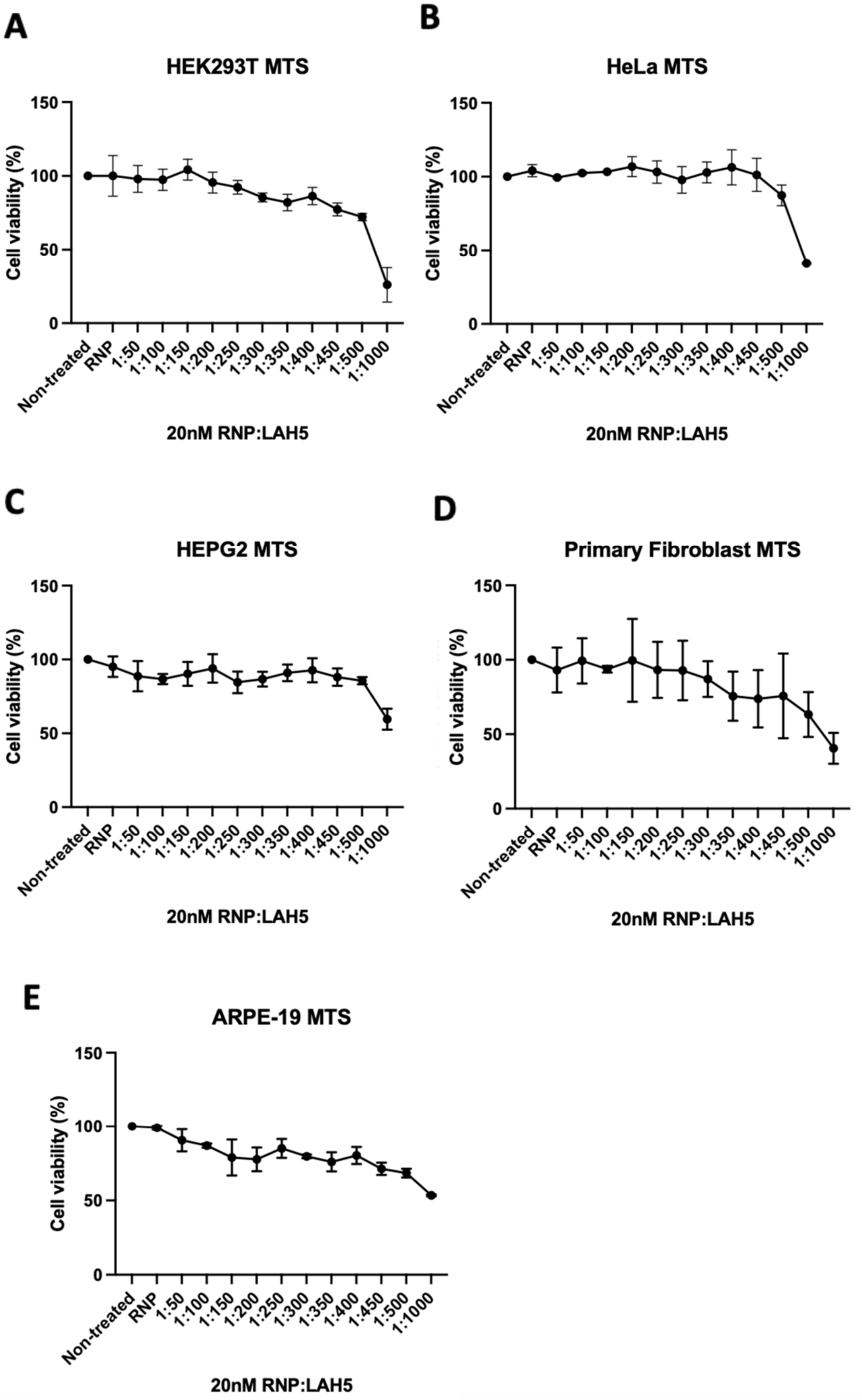

Figure S2. Cytotoxicity study using MTS assay on multiple cell lines: **(A)** HEK293T, **(B)** HeLa, **(C)** HEPG2, **(D)** Primary fibroblasts, **(E)** ARPE-19. RNP/LAH5 nanocomplexes were prepared at 1:50 to 1:1000 M/M ratios using 20nM RNP per well in 96-well plates. The medium was replaced with Opti-MEM containing 100 µl of 20nM RNP complexed with increasing concentrations of LAH5 peptide. MTS assay was performed 24 h after treatment. Non-treated cells were used as a negative control and Triton X-100 (1%) treated cells were used as a positive control. Data are shown as mean ± SD (n = 3).

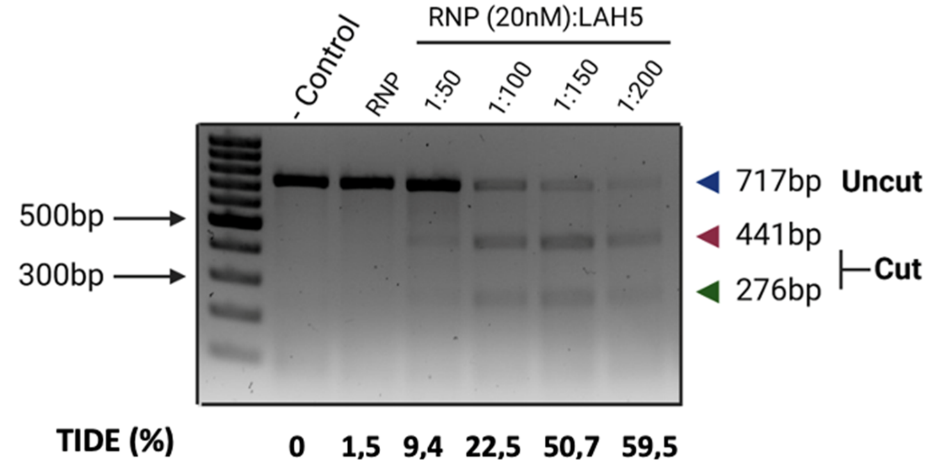

Figure S3. Gene editing efficiency was assessed in ARPE-19 cells, targeting the CCR5 gene. Indel measurements in the *CCR5* locus from RNP/LAH5 treated ARPE-19 cells analyzed by T7E1 (top) and TIDE (bottom) assays (uncut, 717 bp; cut, 441 bp 276 bp; Cas9, 20 nM; CCR5 sgRNA, 20 nM; increasing ratios of LAH5).

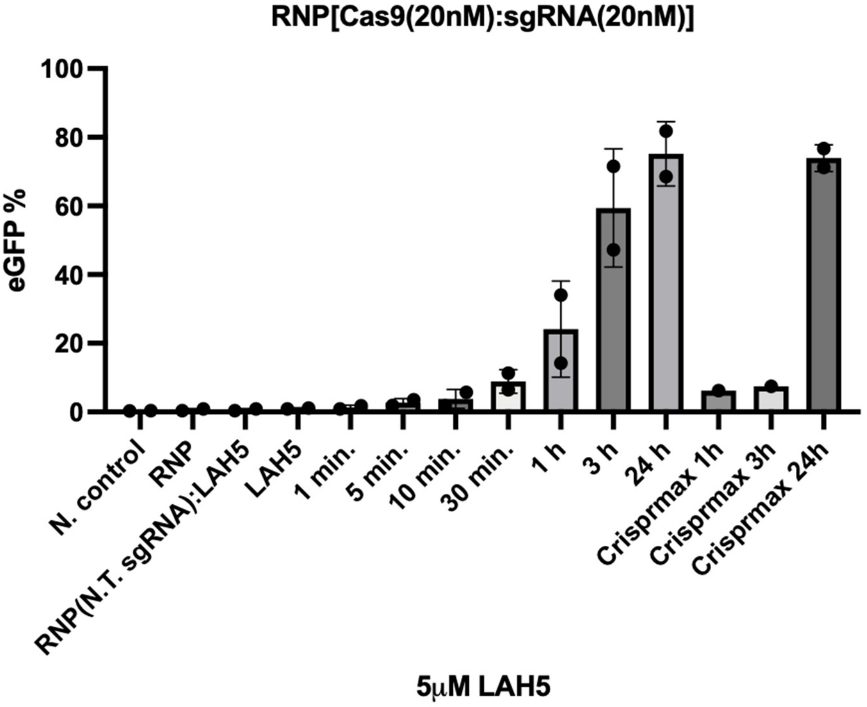

Figure S4. HEK293T stoplight cells were treated with RNP/LAH5 nanocomplexes during different incubation times with 20nM RNP and LAH5 (5µM) peptide. 48h following the transfection, gene editing efficiency (eGFP+ cells) was calculated by flow cytometry. Data are presented as mean ± SD (n = 2)

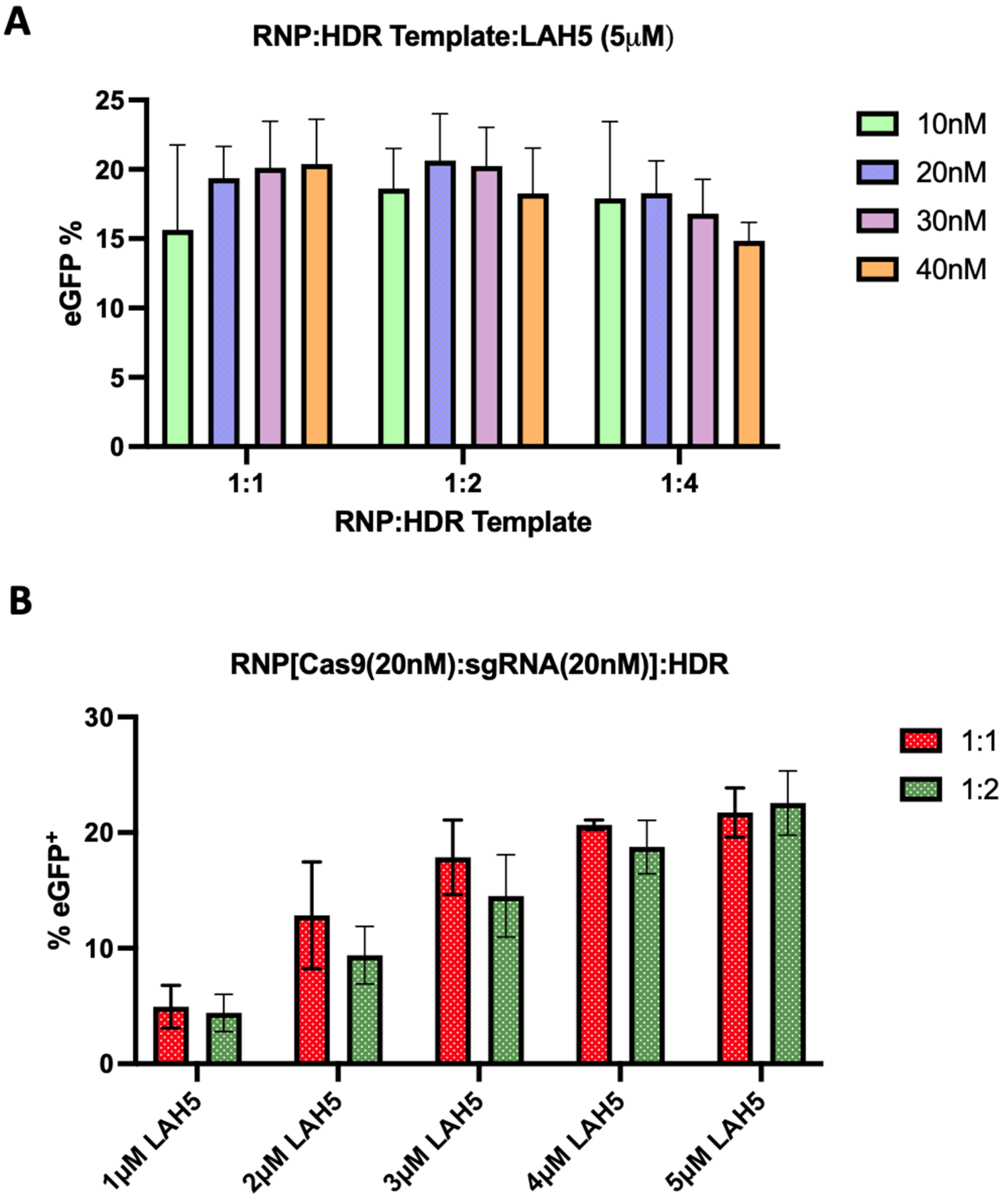

Figure S5. LAH5 peptide-mediated gene correction efficiency using the HDR stoplight reporter system. (**A**) To evaluate the impact of increasing concentrations of RNP/HDR template HEK293T HDR stoplight cells were treated with a range of 10nM to 40nM RNP/HDR template at 1:1, 1:2 and 1:4 ratios. These various RNP/HDR template concentrations were complexed with 5μM of LAH5 peptide. HDR efficiency was quantified by flow cytometry depending on % of eGFP expression (**B)** LAH5 peptide dose-dependent effects on gene correction were tested by treating the cells with RNP/HDR template/LAH5 nanocomplexes, which were prepared using RNP (20nM)/HDR template (20nM) and RNP (20nM)/HDR template (40nM) at an increasing concentration of LAH5 peptide (ranging from 1uM to 5uM). Data presented as mean ± SD (n = 3).

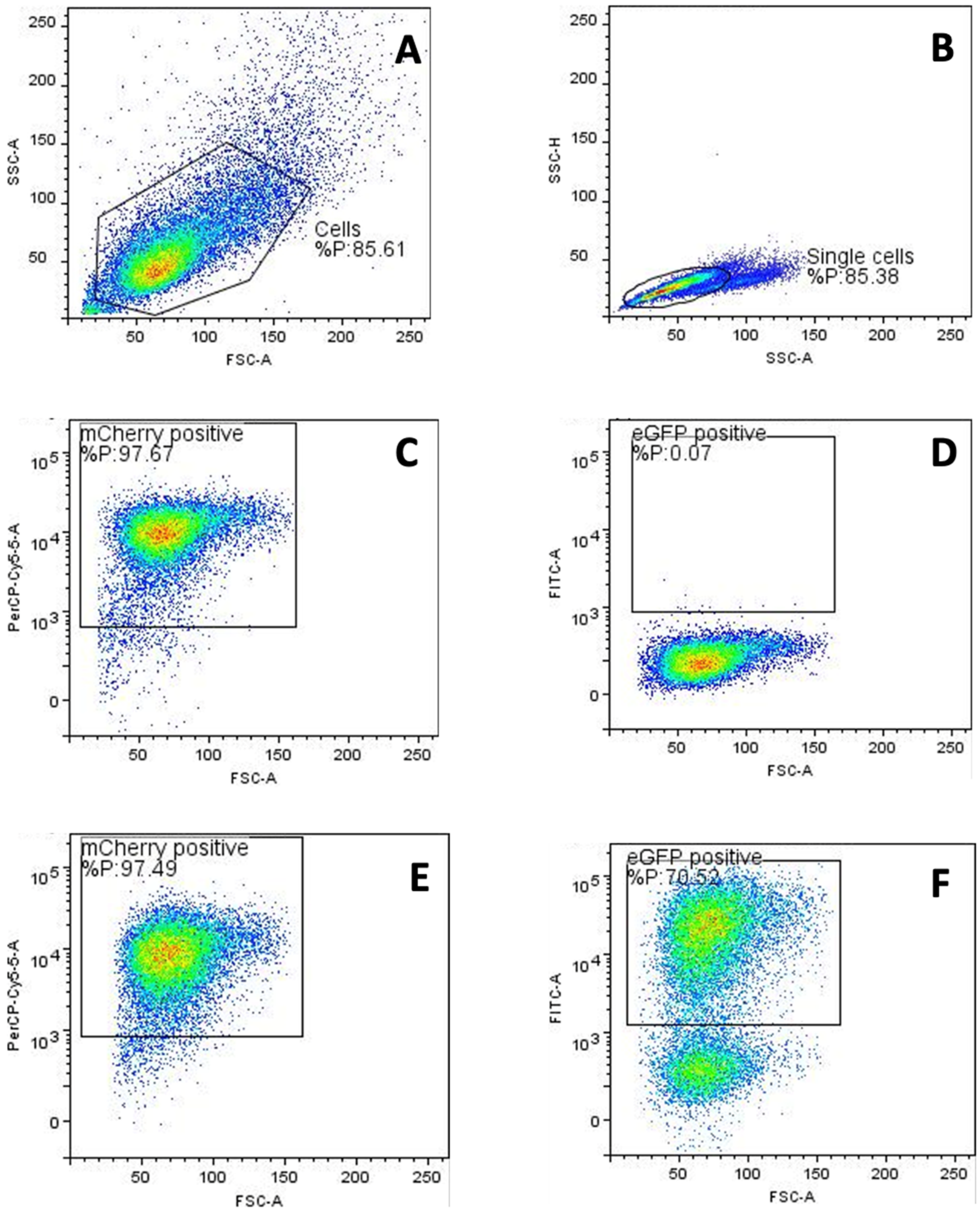

Figure S6. (A) and (B) gating strategy to analyze single cells in flow cytometry experiments using HEK293T reporter cells. Before eGFP analysis, mCherry positive cells are gated using the PerCP-Cy5.5 channel as shown for negative control samples (C) and cells transfected with a 1:200 ratio of RNP/LAH5 nanocomplexes. Within this gate, eGFP positive cells were analyzed using the FITC channel, as show for negative control samples (D) eGFP and cells transfected with a 1:200 ratio of RNP/LAH5 nanocomplexes (F).

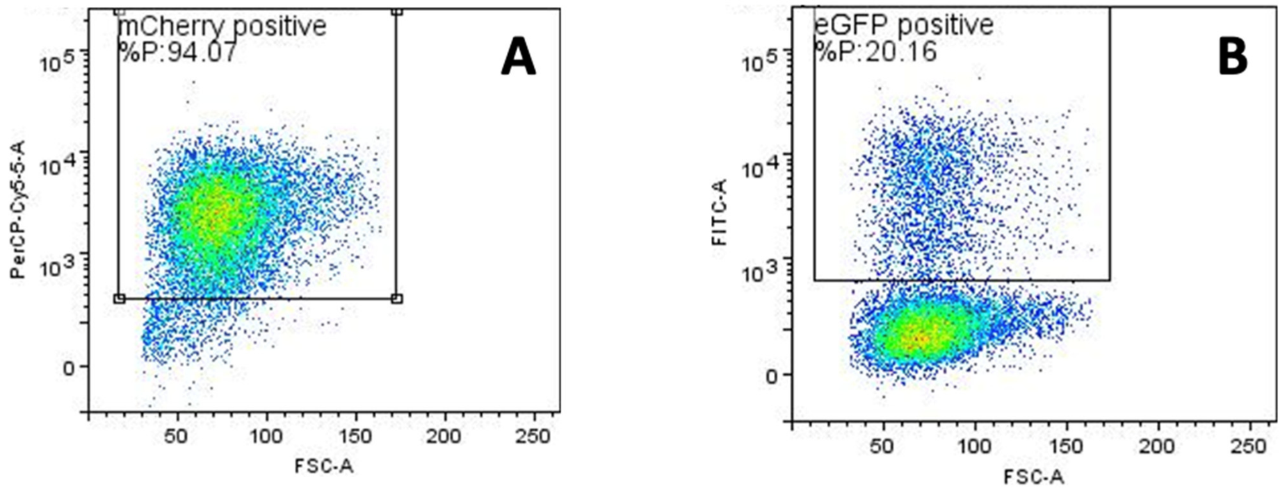

Figure S7. The gating strategy used to analyze HEK293T HDR stoplight cells treated with RNP/HDR template/LAH5 nanocomplexes prepared at 1:1:250 ratio **(A)** selection of mCherry positive population and **(B)** eGFP positive gene-corrected cells.

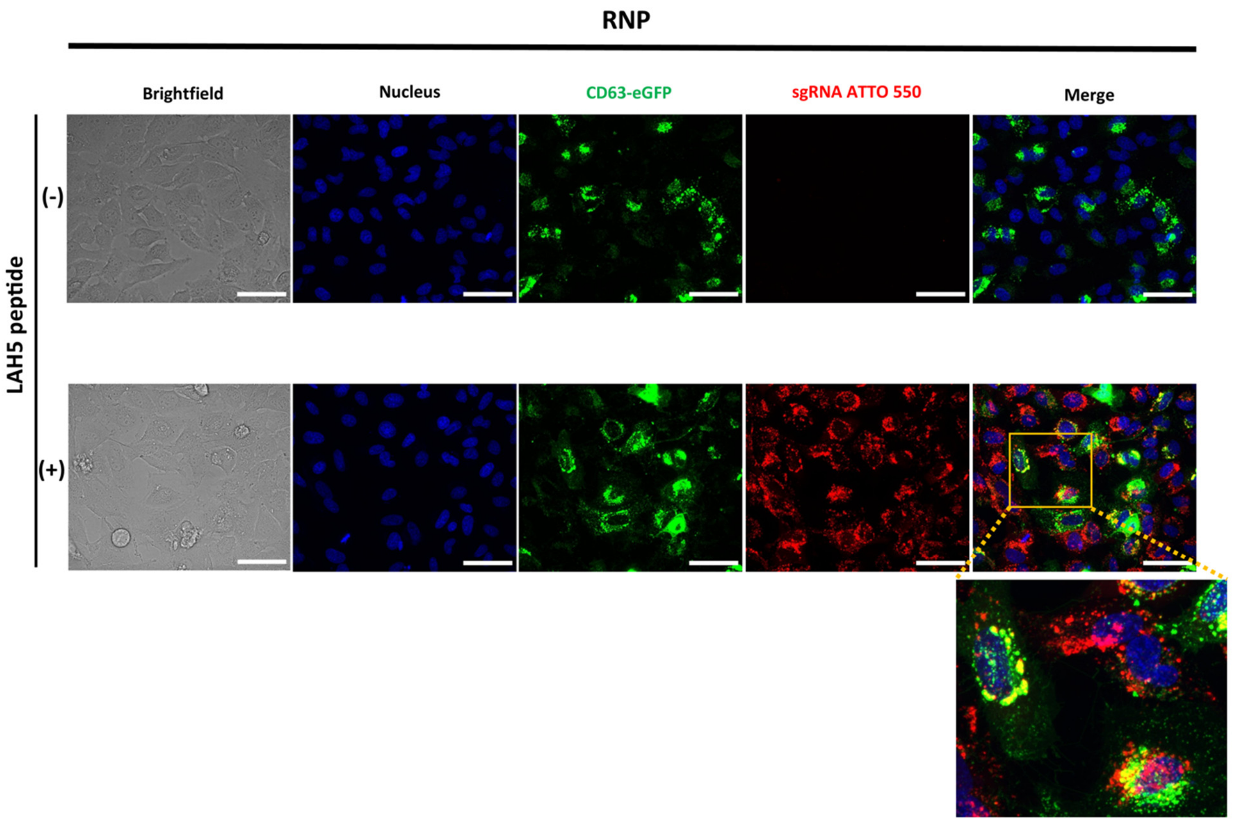

Figure S8. Fluorescence confocal microscopy images depicting the intracellular uptake of Cas9/sgRNA ATTO 550 (RNP), both with- and without LAH5 complexation, in HeLa cells transiently expressing CD63-eGFP. The merged channels demonstrate the co-localization (as indicated in yellow) of the CD63-eGFP late endosome marker and Cas9-sgRNA ATTO 550(RNP)/LAH5 nanocomplexes. No significant uptake of the Cas9/sgRNA ATTO 550 RNPs was observed without LAH5 complexation. Scale bars represent 70 μm.
